# Occupational health risk among healthcare workers during COVID-19 pandemic: actions to limit the risk

**DOI:** 10.1186/s42506-021-00076-z

**Published:** 2021-05-24

**Authors:** Eman Casper

**Affiliations:** Department of Clinical Pharmacy, Faculty of Pharmacy, Ain-Shams University, Cairo, Egypt

**Keywords:** COVID-19, Healthcare workers, Airborne, Morbidity and mortality, Infection prevention, Control measures

## Abstract

The World Health Organization declared coronavirus infection 2019 (COVID-19) as a pandemic in March 2020. The infection with coronavirus started in Wuhan city, China, in December 2019. As of October 2020, the disease was reported in 235 countries. The coronavirus infection 2019 (COVID-19) is a disease with high morbidity and mortality. As of February 2021, the number of confirmed cases of COVID-19 globally is 102,942,987 and 2,232,233 deaths according to WHO report. This infection is caused by severe acute respiratory syndrome coronavirus-2 (SARS-CoV-2), which is a ribonucleic acid (RNA) β-coronavirus. The infection is mainly transmitted through respiratory droplets.

Healthcare workers (HCWs) play an essential role at the front lines, providing care for patients infected with this highly transmittable disease. They are exposed to very high occupational health risk as they frequently contact the infective persons. In order to limit the number of infected cases and deaths among healthcare workers, it is crucial to have better awareness, optimistic attitude, efficient PPE, and adequate health practices about COVID-19.

To the Editor,

The World Health Organization (WHO) declared coronavirus infection 2019 (COVID-19) as a pandemic in March 2020 [1]. The infection with coronavirus started in Wuhan city, China, on December 2019. As of October 2020, the disease was reported in 235 countries [2]. The coronavirus infection 2019 (COVID-19) is a disease with high morbidity and mortality. As of February 2021, the number of confirmed cases of COVID-19 globally is 102,942,987 and 2,232,233 deaths, according to a WHO report [1].

This infection is caused by severe acute respiratory syndrome coronavirus-2 (SARS-CoV-2), which is a ribonucleic acid (RNA) β-coronavirus. The infection is mainly transmitted through respiratory droplets.

Healthcare workers (HCWs) play an essential role at the front lines, providing care for patients infected with this highly transmittable disease. They are exposed to high occupational health risk as they frequently contact the infectious persons. The WHO reported 22,073 cases of COVID-19 among healthcare workers from 52 countries on April 2020 [1].

There are two main sources of healthcare workers’ infection, including workplace and community. In addition, they may be a source of infection for their families and contacts.

This pandemic had exposed the healthcare workers to several mental health problems as they handle the challenges of this pandemic. They may have feelings of guilt or shame. These feelings can affect their ability to perform.

Previous studies had reported the possible risk factors associated with healthcare workers infection. They include late diagnosis of COVID-19 in patients, working in a higher-risk department where large numbers of COVID-19 patients are receiving medical care, longer working hours, sub-optimal adherence and inadequate training to infection prevention and control (IPC) measures such as hand hygiene practices, and lack of or improper use of personal protective equipment (PPE) [3, 4].

Managing these risk factors helps limit the increase in the number of infected cases among healthcare workers. Protecting them is a priority in reducing the burden of the pandemic and providing care to the patients.

In this context, the WHO released recommendations to prevent infection in healthcare settings. All Healthcare workers, who are in close contact of patients with COVID-19, should use contact and droplet precautions. It is also recommended that airborne precautions be performed during procedures and support treatments that include respiratory droplets [5].

The proper use of personal protective equipment (PPE) is critical for the prevention of infection transmission. It is of particular importance to correctly put on and remove PPE during handling the infected persons. Providing continuous training to the healthcare workers is critical to protect them [6].

The risk for COVID-19 infection among healthcare workers is significantly reduced upon the proper and consistent application of the standard precautions. Healthcare workers who are in close contact to infected patients should be periodically screened for COVID-19.

Besides IPC measures, providing psycho-social support to healthcare workers and regular contact to each other to discuss decisions and check on wellbeing is critical to offer safe and decent working environments. After the pandemic is over, it is very important to monitor and continue supporting the healthcare workers [7].

Finally, in order to limit the number of infected cases and deaths among healthcare workers, including physicians, pharmacists, and nurses, in addition to other medical and paramedical staff, it is crucial to have better awareness, optimistic attitude, efficient PPE, and adequate health practices about COVID-19. Governments all over the world should take the responsibility to train all the healthcare members adequately and continuously to be ready for facing the future public health crises.

## Data Availability

Not applicable

## Abbreviations

COVID-19: Coronavirus infection 2019
HCWs: Healthcare workers
IPC: Infection prevention and control
PPE: Personal protective equipment
SARS-CoV-2: Severe acute respiratory syndrome coronavirus-2
WHO: World Health Organization
